# State-of-the-Art Review of Aortic Arch Reconstruction With the Frozen Elephant Trunk

**DOI:** 10.1177/15569845251347968

**Published:** 2025-07-01

**Authors:** Sabin J. Bozso, Ryaan EL-Andari, Rashmi Nedadur, Brandon Loshusan, Holly Smith, Jennifer C. Y. Chung, Jonathan Hong, François Dagenais, Marina Ibrahim, Michael C. Moon, Michael W. A. Chu

**Affiliations:** 1 Division of Cardiothoracic Surgery, University of Alabama at Birmingham, AL, USA; 2 Mazankowski Alberta Heart Institute, University of Alberta, Edmonton, AB, Canada; 3 Division of Cardiac Surgery, Western University, London, ON, Canada; 4 Division of Cardiac Surgery, University of Calgary, AB, Canada; 5 Division of Cardiac Surgery, Department of Surgery, University of Toronto, ON, Canada; 6 Section of Cardiac Surgery, Department of Surgery, University of Manitoba, Winnipeg, MB, Canada; 7 Division of Cardiac Surgery, Institut universitaire de cardiologie et de pneumologie de Québec (IUCPQ), Laval University, QC, Canada; 8 Division of Cardiac Surgery, Montreal Heart Institute, QC, Canada

**Keywords:** aorta, hybrid arch, aortic aneurysm, aortic dissection

## Abstract

Aortic arch replacement operations have undergone substantial evolution with technical advancements, notably the introduction of the frozen elephant trunk (FET) technique. The purpose of this state-of-the-art review is to detail our approach to contemporary aortic arch replacement with FET operations. First, we review the evolution of FET procedures over the years and discuss technical modifications, including cerebral perfusion options, to the aortic arch replacement with FET. We also discuss state-of-the-art technical considerations of head vessel reconstruction and management of the difficult left subclavian artery. We also discuss selected considerations related to the endovascular stent graft component, including landing zone management and when to consider extended distal aortic interventions. We briefly discuss potential complications of which the vigilant clinician should be aware, as well as highlight subtleties in managing aortic dissection compared with aortic aneurysms.

## Graphical abstract

**Visual abstract:**
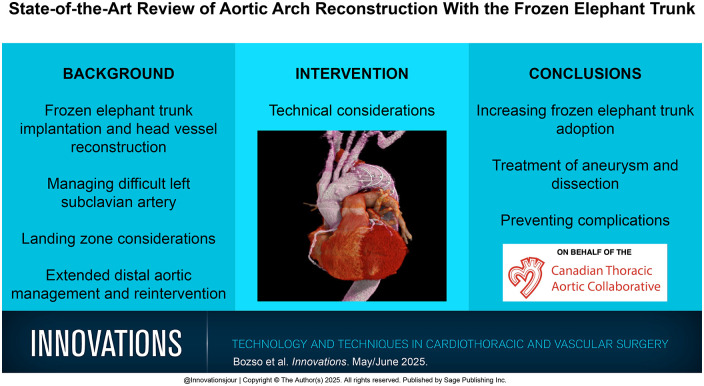

**Central Message**

The FET technique isevolving, with continuous development of novel devices and approaches. We expect this evolution will provide ongoing opportunities for innovation in this rapidly advancing area of aortic surgery.

## Introduction

Aortic arch replacement remains the gold standard treatment for complex aortic arch disease, including acute and chronic aortic dissections, as well as aneurysmal disease. The frozen elephant trunk (FET) approach to total arch replacement combines conventional surgically sewn proximal arch anastomosis with incorporation of a distal endovascular stent graft. This facilitates single-stage management of a wide variety of aortic arch pathologies involving the entire thoracic aorta through a standard sternotomy. Initial experience with FET approaches involved custom-made prostheses; however, commercially available devices to facilitate FET repair are now widely available. As experience grows with this unique approach, many lessons have been learned regarding optimal surgical technique, in addition to management of the distal endovascular stent graft component. In this state-of-the-art review, we describe the evolution of the FET technique and detail nuanced technical considerations for aortic arch replacement with FET devices. We also highlight landing zone and extended distal considerations, including potential complications. Finally, we address the procedural differences and similarities between management of aortic dissection and aortic aneurysm when using the FET approach.

## Evolution of FET Technique

Treatment of the aortic arch and proximal descending thoracic aorta has long posed a surgical challenge. Complex aortic aneurysm or dissection has typically required a multistage approach. Borst and colleagues first introduced the conventional elephant trunk (CET) technique for management of mega-aorta.^1^ The approach involves arch replacement with an extension of the arch prosthesis left “floating” in the proximal descending thoracic aorta. During the second-stage operation, the graft is extended to the desired level as indicated by extent of aortic pathology. Benefits of this approach include a reduced need for proximal dissection and risk to surrounding structures as well as a shorter clamp time during subsequent thoracoabdominal operations.^2^

The development of thoracic endovascular aortic repair (TEVAR) introduced new possibilities for the treatment of aortic disease. Hybrid procedures combining open and endovascular techniques quickly gained popularity, although many still required a staged approach.^3,4^ Karck and colleagues introduced the concept of the FET in 2003.^5^ Antegrade deployment of a stent graft into the proximal descending thoracic aorta at the time of circulatory arrest, followed by arch repair, facilitated a single-stage procedure. This approach has demonstrated safety when compared with CET, in part by removing the risk of mortality between operative stages.^6,7^ Two commonly used stent grafts for this approach include the RelayPro system (Terumo Aortic, Inchinnan, UK) and the GORE TAG device (Gore Medical, Newark, DE, USA). These conventional TEVAR stent grafts are not designed to be deployed antegrade through the curvature of the aortic arch; however, this off-label device used to perform FET repair prevailed until dedicated commercially manufactured, single-piece endoprostheses were developed.

**Fig. 1.**
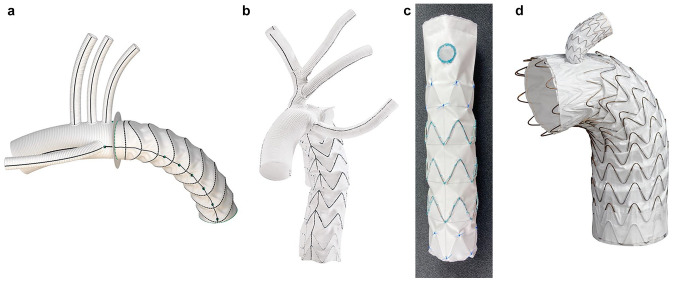
(a) Thoraflex Hybrid Plexus graft (reprinted with permission of Vascutek Limited trading as Terumo Aortic, Inchinnan, UK), (b) E-vita Open Neo trifurcated graft (used with the permission of Artivion, Inc., Kennesaw, GA, USA), (c) FET-FEN graft (courtesy of Cook Medical, Bloomington, IN, USA), and (d) GORE® TAG® Thoracic Branch Endoprosthesis (reprinted with permission from Gore Medical, Newark, DE, USA; see Instructions for Use for complete device information, including approved indications and safety information) for hybrid arch reconstruction.

Several devices have since been introduced and designed to simplify the single-step operation further.^8,9^ Designs vary; however, the uniform concept is a distal rigid stent graft, designed to be deployed antegrade into the distal aortic arch with or without the use of a guidewire. The proximal portion of the device is similar to a conventional aortic arch prosthesis, connected to the distal stent graft with a collar to facilitate the distal anastomosis. Newer devices incorporate epiaortic branches, aiming to bring the surgical anastomosis into zone 2 or even zone 1.^10,11^ The 2 most commonly used hybrid aortic arch grafts are the Thoraflex Hybrid (Terumo Aortic) and the E-vita Open Neo hybrid stent graft system (Artivion, Kennesaw, GA, USA; Fig. 1, Supplemental Video 1). Each of these devices is available in several different configurations, including straight, branched, and trifurcate devices, facilitating whichever arch reconstruction approach the surgeon desires to use. Each of these approaches to aortic arch and head vessel reconstruction will be discussed in the following sections.

## Technical Details of Total Arch Replacement With FET

The sequence of a total arch replacement with FET has been described in 2 general strategies. Following circulatory arrest and removal of the cross-clamp, an arch-first approach involves performing the distal anastomosis, followed by reestablishment of perfusion to the lower body, and then anastomosis of the head vessels (Supplemental Video 2). In contrast, the head-vessel-first technique involves starting with the head vessel anastomoses, reestablishment of head vessel perfusion, and then the distal anastomosis (Supplemental Video 3).^12,13^ Overall, results have improved with time and have been largely similar between the 2 approaches, with much of the variability likely attributable to differences in brain protection, systemic perfusion, and other confounders. Although most centers have a preferred approach, both arch-first and head-vessel-first strategies can be useful in different patient-specific arch anatomy and disease, thus highlighting the usefulness of having both approaches in the surgical toolbox.

**Fig. 2.**
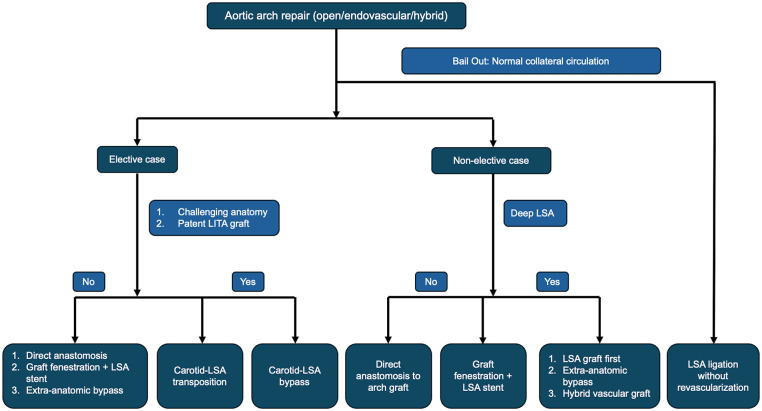
Ishimaru aortic zones.

**Fig. 3.**
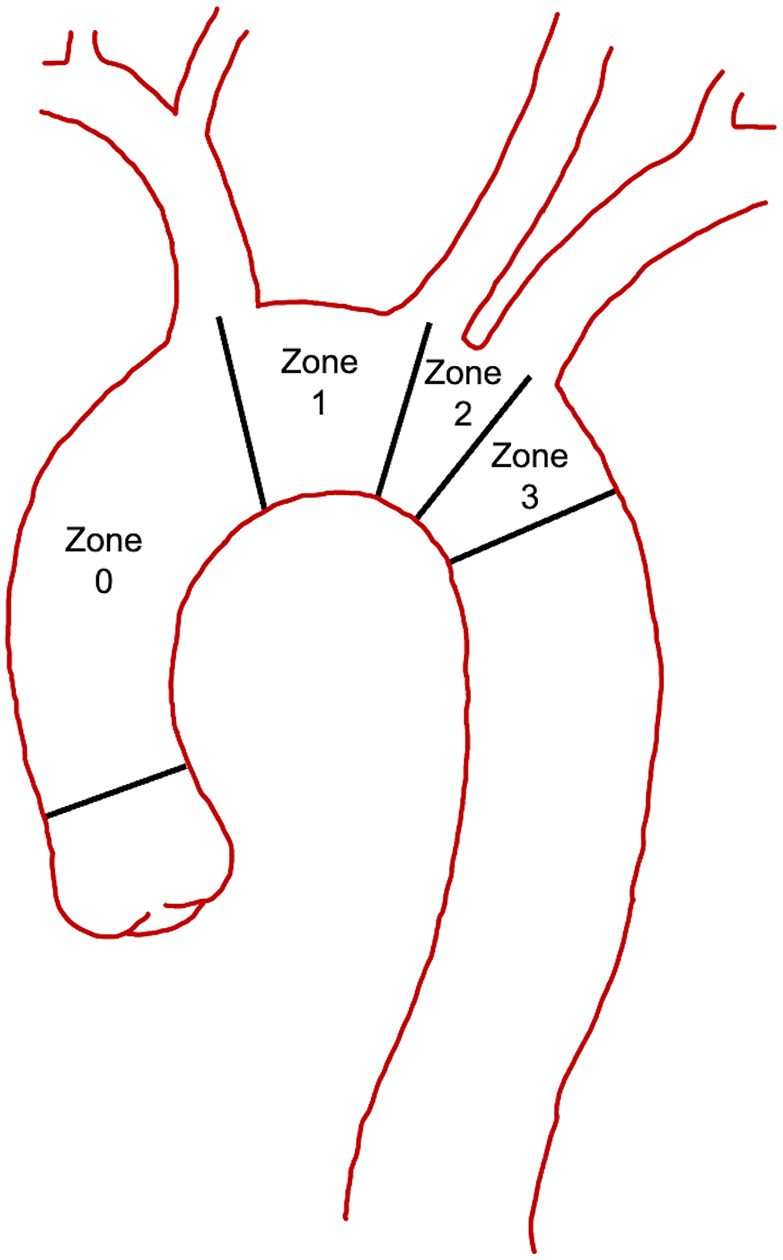
(a) Head vessel reconstruction with multibranched graft, (b) head vessel reconstruction with separate head vessel trifurcated graft, (c) zone 2 arch anastomosis, and (d) zone 0 arch anastomosis with head vessel reconstruction using a trifurcated graft and extra-anatomic left subclavian reconstruction.

The location of the distal anastomosis for a total arch replacement continues to be pushed more proximal from zone 3 toward zone 0 (Fig. 2).^14^ Although a zone 3 distal anastomosis allows for complete replacement of the arch without endovascular intervention, a more proximal anastomosis in zones 0 to 2 does require the addition of an FET to exclude the remaining aortic arch. The distal anastomosis performed at zones 0 to 2 allows for better exposure, minimizes risk to the recurrent laryngeal nerve, and provides an easier distal anastomosis compared with zone 3 while still excluding the entire aortic arch with an FET. Repairing of significant bleeding with a zone 3 distal anastomosis can be very challenging. The head vessels can be managed in various ways including direct anastomoses to a separate branched graft, a hybrid branched or trifurcated graft (Thoraflex or E-vita Neo) that allows for proximalization of the head vessels, the island technique involving anastomosis of the head vessels en bloc to the graft, or a combination of reimplantation of some vessels with anatomic or extra-anatomic bypasses to the remaining vessels (Fig. 3, Supplemental Video 4, Supplemental Video 5).

The technique for the arch anastomosis has traditionally been performed with a running monofilament suture, often in 2 layers. As an alternative, the interrupted anastomosis technique involves using interrupted braided sutures and parachuting the distal anastomosis down in a similar fashion to a valve replacement. This technique is proposed to reduce the difficulty of the distal anastomosis where visualization is challenging by keeping the graft out of the surgical field until it is tied in place.^15^ Because the stent graft portion of the FET can seal the proximal descending aorta, the distal anastomosis is often very hemostatic regardless of which anastomotic technique is used. Felt reinforcement can often be avoided, even in acute aortic dissection.

## Perfusion Considerations

Many options exist for both arterial cannulation and cerebral perfusion when approaching an arch replacement with an FET. Central cannulation involves cannulating the ascending aorta or innominate artery. Peripheral cannulation sites include the axillary, carotid, and femoral arteries. Published evidence has largely found nonsignificant differences in mortality between peripheral cannulation approaches, although recent evidence from large registries has suggested a significant benefit to axillary cannulation with lower rates of stroke in recent studies from the Canadian Thoracic Aortic Collaborative (CTAC) and the International Registry of Acute Aortic Dissection.^16,17^ Right axillary and innominate artery cannulation allow for antegrade cerebral perfusion (ACP) following circulatory arrest via clamping of the proximal innominate artery. Left axillary, femoral artery, and aortic cannulation require direct perfusion through the head vessels for ACP, for example, through a 13F Gundry catheter. Retrograde cerebral perfusion (RCP) is initiated by superior vena cava (SVC) cannulation and providing arterial blood through the snared SVC cannula in a retrograde fashion. A study by the CTAC group found comparable outcomes in a multivariable analysis comparing ACP and RCP strategies.^18^ Carotid cannulation has recently emerged as an alternate cannulation option, allowing for a similar benefit to axillary artery cannulation with antegrade flow to the head vessels without the need for a separate incision.^19^

Temperature management during circulatory arrest has long been debated. A recent randomized controlled trial by Hughes and colleagues randomized patients undergoing arch repair with unilateral ACP to high-moderate (24 to 28 °C), low-moderate (20 to 24 °C), or deep (<20 °C) hypothermia. There was noninferiority between low-moderate and deep hypothermia for postoperative mean global cognitive change, but verbal memory was better preserved in the deep hypothermia group.^20^ Blood loss was higher in the deep hypothermia group, with otherwise comparable adverse events. Although intraoperative decisions such as cannulation, cerebral perfusion, and degree of hypothermia are currently made on a case-by-case basis by the primary surgeon, machine learning models have recently demonstrated the ability to identify predictors of positive or adverse outcomes and may have a future role for intraoperative decision-making.^21^

## Head Vessel Reconstruction

Management of the innominate artery, the left common carotid artery, and left subclavian artery (LSA) is a major component of operative planning that has a large impact on the technical success of FET repair. Revascularization of the arch branch vessels during arch reconstruction may be performed anatomically using the island technique. The arch branch vessels are separated as an island and reattached directly to the graft using a single anastomosis. Although simple in concept and a useful technique, even in cases of aortic dissection, it has largely fallen out of favor because of some recognized disadvantages.^22^ The posterior aspect of this island anastomosis is difficult to repair if there is any bleeding. Young patients with or without connective tissue disease may have significant amounts of unresected abnormal aortic tissue. The tissue may be subject to further dilation and future reintervention. In older patients with atherosclerosis, plaque burden tends to be in the arch and arch branch origins. Manipulating and suturing around this region may cause plaque disturbance.

More commonly, the arch branch vessels are individually transected just beyond their origin in areas with less atherosclerotic burden and anastomosed separated onto smaller grafts. Hybrid FET devices have configurations that include 3 prefabricated branches for this purpose. With this technique, the distal anastomosis can be performed first, and the head vessels are then reconstructed in reverse order from subclavian, carotid, and innominate, or a previous head vessel graft can be attached (below). Separate head vessel reconstruction is much more hemostatic and enables all abnormal aortic tissue to be excluded.^23^

If one desires arch debranching first to allow earlier bilateral ACP, then multiple grafts are available to facilitate this by providing trifurcate branching for the innominate artery, left common carotid artery, and LSA. The proximal portion of this graft is then attached to the arch or ascending aortic graft. This type of arch debranching commonly brings the arch branch vessels more proximally. This is desirable if future arch TEVAR is planned as part of a staged approach, rather than direct use of the hybrid arch devices.^24^

## Management of the Difficult LSA

The management of the difficult LSA continues to be challenging in aortic arch surgery due to its deep and lateral position within the chest when approached from a sternotomy. One can predict its difficulty on the computed tomography scan by assessing its position relative to the trachea, where an LSA take off posterior to the trachea could be considered more challenging. In addition, the quality of the LSA could be poor or compromised due to dissection, aneurysm, calcium, or scar from previous intervention. The best visualization of the vessel is often during circulatory arrest, and thus, its management prolongs the circulatory arrest time.^25^

**Fig. 4.**
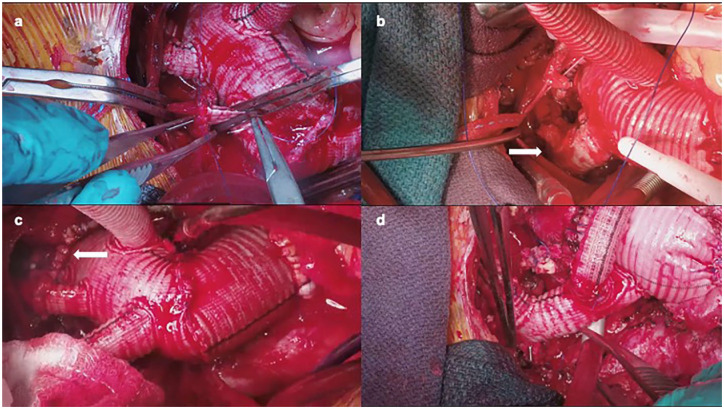
Flowchart describing aortic arch reconstruction options. LITA, left internal thoracic artery; LSA, left subclavian artery.

In an elective setting, the goal should be to achieve revascularization of the LSA to reduce the risk of neurologic complication, spinal cord injury, and limb ischemia.^26,27^ Options for revascularization include anatomic and extra-anatomic approaches and many evolving hybrid strategies (Fig. 4). Anatomic surgical approaches include zone 3 distal arch anastomosis with direct anastomosis of the LSA to a trifurcate graft, which is the most technically challenging of the options. Transthoracic aorta-axillary extra-anatomic bypass with ligation of the subclavian origin is another approach that allows for a simpler LSA anastomosis and a zone 2 distal aortic anastomosis but necessitates another surgical site and requires a tract in the chest wall that can be prone to kinking and external compression.^28^ Extra-anatomic reconstructions include LSA to left carotid transposition or bypass with proximal plug/ligation with near equivalent results with both approaches. There is increased association of vocal cord palsy with transposition, and it can be difficult in patients with patent internal mammary grafts.^29^ There are several hybrid strategies that have also become popularized such as the branched stented anastomosis FET repair (B-SAFER) or the zone 2 arch with single-branch TEVAR completion.^6,7^ Other innovative solutions are modular hybrid^10,30^ vascular grafts with a stent graft portion that deploys into the LSA and a Dacron portion that can be surgically anastomosed.^31,32^ More recently, the Cook Hybrid graft (Cook Medical, Bloomington, IN, USA) has incorporated a reinforced fenestration allowing for a reliable and reproducible technique to revascularize the LSA.

In extremis, it might be necessary to ligate or sacrifice the LSA, and these patients are at high risk of neurologic injury, spinal cord ischemia (SCI), and limb ischemia. In these cases, patients should be closely monitored for signs of ischemia, and prompt revascularization in the event of injury can possibly reverse and prevent further complication.^27^

## Landing Zone Considerations

Thus far, we have mainly discussed the proximal and head vessel reconstruction of the FET procedure. One of the unique and defining features of these hybrid grafts is the presence of a covered stent graft at the distal end. The characteristics of this stent graft related to the distal landing zone and extent of coverage are the next topics that will be explored. First, the planning of an FET procedure should be discussed within a multidisciplinary aortic team. There are 3 characteristics of the distal landing zone to consider: (1) type of FET stent graft, (2) diameter, and (3) length. There are no comparative data to provide guidance on which device to use, and this is primarily dictated by surgeon preference. The Thoraflex hybrid graft has a nitinol-ringed stent design that exerts less radial force in the main body of the FET with additional reinforcement at the distal-most stent, which theoretically may conform better in the angulated aortic arch or proximal descending thoracic aorta. The E-vita Neo, Cook Hybrid, and hand-sewn, off-label FET devices use a Z-stent design that provides consistent radial force throughout its entire length, which may be beneficial to prevent graft infoldings particularly in compressed true lumens in chronic aortic dissection pathology. The FET procedure can treat proximal thoracic aortic pathology in a single stage. Kandola and colleagues found in their retrospective analysis that a >10% distal stent oversize and >30 mm sealing length minimized endoleak and reintervention.^33^ In principle, because the proximal hybrid FET graft is surgically sewn, the distal landing zone requirements may not have to be as robust as traditionally described with standard endovascular grafts.

Nonetheless, distal landing zones that are angulated, tortuous, atheromatous, or calcified will require longer sealing lengths. As FET grafts are deployed under circulatory arrest, without fluoroscopic guidance, precise implantation is lacking, and careful interrogation of preoperative imaging and good wire control can help to predict the location of the distal placement of the FET. FET thrombus formation has been described but is poorly understood. Some have hypothesized that early intraluminal thrombus formation may occur in free-floating FET devices that are not sealed distally due to stent-graft “pockets” around the aortic arch inner curve.^34^ This may be pronounced in larger FET diameters with excess fabric and the potential for deeper “pockets.”^34^ Thus, some centers support the selection of smaller diameter FET in extensive thoracic pathology where the FET is not sealed distally. Finally, the length of the device should be considered and balanced between longer seal zones to prevent endoleak and the risk of spinal cord malperfusion. The length selected also depends on the proximal placement of surgical anastomosis. A zone 0 or 1 surgical anastomosis will require longer stent lengths compared with a zone 2 or 3 surgical anastomosis. A meta-analysis identified that a stent length of >15 cm and one that went beyond T8 had a higher risk of spinal cord malperfusion.^35^ Ideally, the FET device will be placed beyond the curvature of the distal aortic arch but proximal to T8 or large intercostal arteries seen on imaging that would be important to preserve.

## Extended Distal Aortic Management and Reintervention

Distal reintervention following FET procedures has been reported in up to 64% of cases at 3 years.^36^ Indications for reintervention may be secondary to a planned second-stage completion, owing to initial extensive aortic disease, distal disease progression, or to FET-induced distal complication.

In rare cases of mega-aorta with contained rupture distal to the FET landing site, a planned emergent second-stage procedure is required. In such circumstances, the FET procedure should be conducted in a hybrid suite and distal TEVAR completion performed at the index FET operation. More often, the planned elective second-stage completion should be deferred 2 to 4 weeks after FET to ensure adequate spinal cord collateral circulation. In cases of aneurysmal disease with adequate distal neck, TEVAR extension can be carried out with low risks and good outcomes.

In diffuse thoracoabdominal aneurysmal disease, the strategy should aim, when feasible, to convert an extent II repair to an extent IV repair by extending the FET with a TEVAR to the aortic hiatus. A third-stage endovascular completion is then carried out. In situations in which an open approach is indicated, patient-specific anatomy can help to dictate whether further endovascular extension can help to ease reconstruction or whether the thoracoabdominal graft should just extend from the FET. Most hybrid FET grafts can be clamped or controlled with an occlusion balloon and can be sewn to easily with little challenge. Some prefer to reinforce that anastomosis, particularly if there is a major size discrepancy.

In addition to the previous recommendations, specific considerations should be given to the chronically dissected thoracoabdominal aneurysms. The Knickerbocker and septectomy techniques are proposed to create a full-circumference aortic landing zone, whereas the candy plug technique is designed to occlude retrograde flow within the thoracic aortic false lumen.^37,38^ Refinement in indications, techniques, and outcome analysis will further delineate the role of these techniques in a chronic dissection setting.

## Complications

Although the FET procedure has significantly improved patient outcomes, it is associated with several potential complications. Neurologic complications such as permanent stroke and SCI are the most devastating complications of aortic surgery. A meta-analysis of more than 3,154 patients found that the rate of SCI was 4.7% and the rate of stroke was 7.6%.^35,39^ The risk of SCI increases with the length of the descending aorta covered or those with previous aortic repair. Graft-related thromboembolic events such as thrombus within the FET stent graft or an embolic event can also occur, leading to distal embolization and potential organ damage and death. One study found that 11.7% of patients had a thromboembolic complication before hospital discharge.^40^ Early monitoring and intervention are key to prevention.

Another significant complication is distal stent graft–induced new entry tears (SINE), in which the distal part of the graft induces new tears, complicating outcomes and requiring additional interventions. Kreibich et al. found that these new tears were particularly problematic in patients with extended aortic involvement.^41^ Endoleaks, particularly type Ib, remain a significant risk after FET, often due to inadequate graft sealing, as noted by Kandola et al.^33^ Authors recommend >10% distal stent oversize and >30 mm sealing length to minimize endoleak and reintervention and increasing multidisciplinary collaboration with endovascular surgeons to improve distal stent planning.

Despite advancements in technique, mortality remains a concern, especially in high-risk patients, with Ouzounian et al. reporting mortality rates linked to surgical complexity and comorbidities.^42^ In addition, recurrent laryngeal nerve injury, which can cause hoarseness and airway complications, is a potential complication, often from direct trauma during surgery or postoperative swelling around the aortic arch.

## Aneurysm Versus Dissection

There are several important differences that exist between treating aortic aneurysm and aortic dissection with an FET approach, including both preoperative and intraoperative considerations. In general, most clinicians advocate that when treating aortic aneurysm, the stent graft portion of the FET should be approximately 10% to 20% oversized.^43,44^ This allows for an adequate seal distally to prevent type 1b endoleak and subsequent aneurysm sac filling. In an acute aortic dissection, oversizing should be avoided with a nominal 0%, to maximum 10%, oversizing. Often, we will choose the smallest graft size available in acute DeBakey I aortic dissection because the entry tear is resected and the purpose of the stent graft portion Is to gently encourage remodeling and providing a future endovascular landing zone. Oversizing in acute dissection and in those with known or suspected connective tissue disorders carries the risk of aortic rupture and can lead to distal SINE. The shortest graft length necessary to exclude the entry tear in a dissection or allow for adequate sealing in an aneurysm should be chosen.

Sizing the FET device in type B aortic dissection can be nuanced, and there is no consensus as to which strategy is best. In acute type B aortic dissection with retrograde extension that requires a total arch replacement, some size the stent graft portion of the FET device using the total aortic diameter, based on the concept that in the acutely dissected aorta, there has not been time for a rigid septum to form, and the goal should be to allow full true lumen expansion and false lumen thrombosis. In contrast, many others size the FET to the maximum dimension of the true lumen, with the assumption to allow slow, progressive remodeling and not to increase the risk of distal SINE.

There is a growing interest in using FET devices in acute type A aortic dissections.^45^ Common indications to consider this approach in the acute type A dissection setting are the presence of an arch aneurysm or arch entry tear, concomitant descending thoracic aneurysm or contained rupture, distal malperfusion, young patients, and those with known connective tissue disorders.^46^ There are no prospective trials on FET repair compared with hemiarch alone in acute type A aortic dissections; therefore, decision making is based on nonrandomized clinical outcomes data, consensus guidelines, and surgeon preference.^47^ Randomized evidence is greatly needed to help address the question of hemiarch versus extended distal repair in acute type A aortic dissection. The TITAN:HEADSTART trial (NCT03885635) is enrolling patients to evaluate this question and follow patients with clinical and radiographic follow-up for up to 3 years.^48^ A guidewire is typically encouraged when treating dissected aorta to ensure that the FET device is delivered with precision into the true lumen. Femoral arterial access is obtained under ultrasound, and a stiff wire is advanced into the aortic arch under transesophageal echocardiography guidance. Intravascular ultrasound can also be used to confirm true lumen placement and identify any major fenestrations or reentry tears. Deployment over a guidewire is especially important if complex reentry tears exist in the aortic arch or proximal descending aorta, a common occurrence in chronic arch dissection.

## Conclusions

Aortic arch replacement operations have undergone substantial evolution with major technical modifications, notably the introduction of the FET approach. The evolution of the FET technique is ongoing, with novel devices and approaches in continuous development. Much of the refinement will be directed toward minimizing the upfront surgical risk with new devices that simplify arch reconstruction and reduce potential aortic reinterventions. Future research regarding both short-term and long-term outcomes in unique groups of patients including both younger and older patients, acute and chronic aortic dissections, and those with connective tissue disorder is necessary and ongoing. The adoption of the FET approach to aortic arch reconstruction is growing, as is the population of patients who are candidates for this approach. As such, we expect current surgical techniques will continue to evolve, providing ongoing opportunities for innovation in this rapidly advancing area of aortic surgery.
